# Dual X-ray- and Neutron-Shielding Properties of Gd_2_O_3_/NR Composites with Autonomous Self-Healing Capabilities

**DOI:** 10.3390/polym14214481

**Published:** 2022-10-22

**Authors:** Worawat Poltabtim, Arkarapol Thumwong, Ekachai Wimolmala, Chanis Rattanapongs, Shinji Tokonami, Tetsuo Ishikawa, Kiadtisak Saenboonruang

**Affiliations:** 1 Department of Applied Radiation and Isotopes, Faculty of Science, Kasetsart University, Bangkok 10900, Thailand; 2 Institute of Radiation Emergency Medicine, Hirosaki University, Aomori 0368564, Japan; 3 Special Research Unit of Radiation Technology for Advanced Materials (RTAM), Faculty of Science, Kasetsart University, Bangkok 10900, Thailand; 4 Department of Materials Science, Faculty of Science, Kasetsart University, Bangkok 10900, Thailand; 5 Polymer PROcessing and Flow (P-PROF) Research Group, Division of Materials Technology, School of Energy, Environment and Materials, King Mongkut’s University of Technology Thonburi, Bangkok 10140, Thailand; 6 Department of Radiation Physics and Chemistry, Fukushima Medical University, Fukushima 9601295, Hikarigaoka, Japan; 7 Kasetsart University Research and Development Institute (KURDI), Kasetsart University, Bangkok 10900, Thailand; 8 Specialized Center of Rubber and Polymer Materials in Agriculture and Industry (RPM), Faculty of Science, Kasetsart University, Bangkok 10900, Thailand

**Keywords:** natural rubber, Gd_2_O_3_, self-healing, shielding, mechanical properties, X-rays, neutrons

## Abstract

The neutron- and X-ray-shielding, morphological, physical, mechanical, and self-healing properties were investigated for natural rubber (NR) composites containing varying gadolinium oxide (Gd_2_O_3_) contents (0, 25, 50, 75, and 100 parts per hundred parts of rubber; phr) to investigate their potential uses as self-healing and flexible neutron- and X-ray-shielding materials. Gd_2_O_3_ was selected as a radiation protective filler in this work due to its preferable properties of having relatively high neutron absorption cross-section (σ_abs_), atomic number (Z), and density (ρ) that could potentially enhance interaction probabilities with incident radiation. The results indicated that the overall neutron-shielding and X-ray-shielding properties of the NR composites were enhanced with the addition of Gd_2_O_3_, as evidenced by considerable reductions in the half-value layer (HVL) values of the samples containing 100 phr Gd_2_O_3_ to just 1.9 mm and 1.3 mm for thermal neutrons and 60 kV X-rays, respectively. Furthermore, the results revealed that, with the increase in Gd_2_O_3_ content, the mean values (± standard deviations) of the tensile strength and elongation at break of the NR composites decreased, whereas the hardness (Shore A) increased, for which extreme values were found in the sample with 100 phr Gd_2_O_3_ (3.34 ± 0.26 MPa, 411 ± 9%, and 50 ± 1, respectively). In order to determine the self-healing properties of the NR composites, the surfaces of the cut samples were gently pressed together, and they remained in contact for 60 min; then, the self-healing properties (the recoverable strength and the %Recovery) of the self-healed samples were measured, which were in the ranges of 0.30–0.40 MPa and 3.7–9.4%, respectively, for all the samples. These findings confirmed the ability to autonomously self-heal damaged surfaces through the generation of a reversible ionic supramolecular network. In summary, the outcomes from this work suggested that the developed Gd_2_O_3_/NR composites have great potential to be utilized as effective shielding materials, with additional dual shielding and self-healing capabilities that could prolong the lifetime of the materials, reduce the associated costs of repairing or replacing damaged equipment, and enhance the safety of all users and the public.

## 1. Introduction

As the demand for greener technologies has rapidly increased in recent years following the Sustainable Development Goals (SDGs) introduced by the United Nations (UN) [1], radiation technologies have become one of the most sought-after tools to satisfy such demands due to their reduced use of hazardous chemicals during irradiation and procedures, adaptability to large-scale production, and vast range of applications, such as the determination of transfer mechanisms for minerals and radionuclides in plants [2,3], non-destructive imaging for cultural heritage artifacts [4], diagnostic and radiotherapy purposes for brain and breast cancers [5,6], measurement of moisture in soils [7], and gemstone modification [8]. However, despite their acknowledged benefits, excessive exposure to different types of radiation, especially those from neutrons and X-rays, can harmfully affect users and the public, possibly resulting in permanent injuries or deaths [9].

To minimize the risk of potential adverse effects from excessive radiation exposure, suitable and effective radiation-shielding equipment must be implemented in all nuclear-related facilities following a radiation safety concept, namely As Low As Reasonably Achievable, or ALARA [10]. Generally, the selection of the main materials and radiation-protective fillers used to produce radiation-shielding equipment depends on several factors, such as the type and energy of the incident radiation, as well as the physical and mechanical requirements for the intended applications. For example, to attenuate thermal neutrons (neutrons with an energy of 0.025 eV), compounds containing elements with a high neutron absorption cross-section (σ_abs_), such as boron (B), boron carbide (B_4_C), and boron oxide (B_2_O_3_), are often used due to the relatively high σ_abs_ value of B (^10^B has a σ_abs_ value of 3840 barns, while ^nat^B has a value of 768 barns) [11], which considerably enhances the absorption probabilities between incident thermal neutrons and the material. On the other hand, for X-ray attenuation, materials consisting of heavy elements or compounds, such as lead (Pb), lead oxide (PbO), bismuth oxide (Bi_2_O_3_), tungsten oxide (WO_3_), and barium sulfate (BaSO_4_), are commonly implemented due to the relatively high atomic numbers (Z) of Pb, Bi, W, and Ba (Z = 82, 83, 74, and 56, respectively), as well as the high densities (ρ) of Pb, PbO, Bi_2_O_3_, WO_3_, and BaSO_4_ (ρ = 11.3, 9.5, 8.9, 7.2, and 4.5 g/cm^3^, respectively) [12,13,14], which considerably enhance the interaction probabilities between incident X-rays and the material through two main mechanisms, namely photoelectric absorption and Compton scattering, subsequently resulting in improved X-ray-shielding properties of the composites [15].

While the use of these fillers can noticeably improve the radiation attenuation capabilities of materials, the lack of dual shielding properties (that is, the ability to effectively and simultaneously attenuate both thermal neutrons and X-rays) has resulted in the need to either acquire two distinct types of shielding materials or to mix two different fillers in the same material [16,17]. While these methods are possible, they could potentially increase the cost and space requirements to accommodate thicker materials, as well as possibly reducing desirable mechanical and physical properties of the shielding materials due to particle agglomeration from having filler contents that are too high [13]. To alleviate such drawbacks, gadolinium oxide (Gd_2_O_3_), which is a rare-earth compound, has drawn much attention from researchers and product developers in radiation safety due to the high values of σ_abs_ (49,700 barns) and Z (64) for Gd, as well as the high ρ of Gd_2_O_3_ (7.4 g/cm^3^), which result in its ability to simultaneously attenuate both thermal neutrons and X-rays. Some examples of Gd_2_O_3_ used as radiation protective filler are the development of neutron-shielding hydrogels from poly(vinyl) alcohol (PVA), which indicated substantial enhancements in the ability of the hydrogels to attenuate thermal neutrons after the addition of Gd_2_O_3_. This was evidenced by the half-value layer (HVL; the thickness of a material that can attenuate 50% of the initial intensity of radiation), which were reduced from 146.3 mm in a pristine PVA hydrogel to just 3.6 mm in a 10.5 wt% Gd_2_O_3_/PVA hydrogel. Subsequently, this shielding improvement reduced space requirements to accommodate the materials by almost 40-fold [18]. Another work on the use of Gd_2_O_3_ by Kaewnuam et al. investigated the gamma-shielding properties of WO_3_-Gd_2_O_3_-B_2_O_3_ glass and showed that the HVL values of the glasses were reduced from 1.424 cm in a sample with 17.5 wt% Gd_2_O_3_ to 1.326 cm in a sample with 27.5 wt% Gd_2_O_3_ determined based on 662 keV gamma rays emitted from ^137^Cs [19]. These two examples clearly show the shielding effectiveness of Gd_2_O_3_ for both thermal neutron and high-energy photon attenuations and present the advantages of Gd_2_O_3_ as a radiation-protective filler in comparison to common Pb, Bi, and B compounds.

Another important factor to consider in producing radiation-shielding materials is the selection of the main matrix, for which the selection largely depends on the requirements of the intended applications. For example, applications requiring high flexibility, strength, and elongation usually rely on natural rubber (NR) or synthetic rubber (SR). For example, B_2_O_3_/NR [11], B_4_C/NR [20], and H_3_BO_3_/ethylene-propylene diene monomer (EPDM) [21] composites have been developed for use as flexible, neutron-shielding materials, while Bi_2_O_3_/EPDM [13], WO_3_/EPDM [13], BaSO_4_/NR [14], and Pb/NR [22] composites have been utilized as flexible, X-ray-shielding and gamma-shielding materials. While these composites could serve their mandatory purpose (the ability to attenuate incident thermal neutrons or X-rays (depending on filler type) with high flexibility and strength), the lack of self-healing capabilities in most common NR and SR composites has resulted in extra procedures or new materials needed to restore full function once the materials are damaged, inevitably shortening their lifetimes and increasing operational costs. To resolve these shortcomings, Xu et al. successfully developed autonomously self-healing NR composites by introducing controlled peroxide-induced vulcanization to generate ionic cross-links to NR networks via the polymerization of zinc dimethacrylate (ZDMA), which slowed the formation of non-reversible covalent cross-links while generating a reversible ionic supramolecular network to NR, enabling the ability to autonomously heal after damage [23,24]. Hence, to expand their usefulness to other applications, the concept of autonomously self-healing NR materials can be adapted for the production of radiation-shielding materials, which could not only present the mentioned benefits but also improve safety for radiation users from damaged equipment.

Therefore, this current work investigates the properties of flexible Gd_2_O_3_/NR composites for their potential use as dual thermal-neutron- and X-ray-shielding materials with autonomously self-healing capabilities by introducing reversible ionic supramolecular cross-links to NR networks. In order to understand the effects of the Gd_2_O_3_ fillers on the properties of the composites, the Gd_2_O_3_ contents were varied with values of 0, 25, 50, 75, and 100 parts per hundred parts of rubber (phr) by weight to thoroughly investigate the properties of interest, which consisted of thermal-neutron and X-ray-shielding properties (based on the linear attenuation coefficient (µ), the mass attenuation coefficient (µ_m_), the half-value layer (HVL), the tenth-value layer (TVL), and the Pb equivalence (Pb_eq_)), as well as mechanical (based on tensile strength, elongation at break, and hardness (Shore A) both before and after self-healing), morphological, and physical (based on density) properties. The outcomes of this work can not only present valuable information on the dual neutron- and X-ray-shielding properties of the developed Gd_2_O_3_/NR composites, but may also offer a novel procedure to obtain self-healing NR composites that is beneficial for the future development of other radiation-shielding products.

## 2. Experimental

### 2.1. Materials and Chemicals

**Figure 1:**
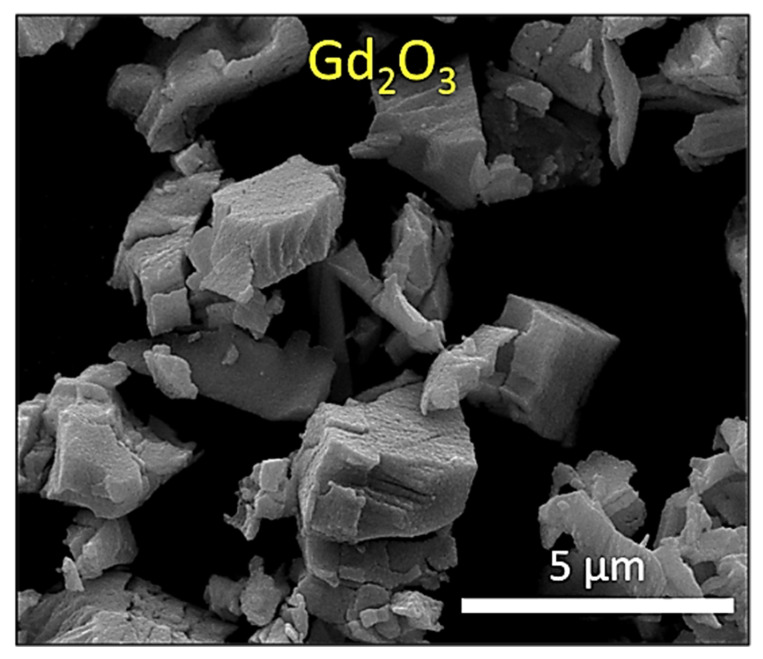
SEM image of Gd_2_O_3_ particles used in this work.

Natural rubber (STR 5CV) with a Mooney viscosity of 60.8 (at 100 °C) was supplied by Hybrid Post Co., Ltd. (Bangkok, Thailand). The names, contents, roles, and suppliers of the chemicals used for sample preparation are shown in Table 1. An image of Gd_2_O_3_ powder captured using a scanning electron microscope (SEM; Quanta 450 FEI: JSM-6610LV, Eindhoven, the Netherlands) is shown in Figure 1, which indicates that the average particle size of the Gd_2_O_3_ powder was 3.4 ± 0.4 µm, as determined using ImageJ software version 1.50i (Bethesda, MD, USA).

### 2.2. Sample Preparation

The NR samples were prepared using two steps: mastication and then compounding. Initially, the NR was masticated on a two-roll mill (R11-3FF, Kodaira Seisakusho Co., Ltd., Tokyo, Japan) for 5 min. Then, the masticated NR was compounded with the chemicals (Table 1) for a further 15–20 min. Notably, although the content of Gd_2_O_3_ was as high as 100 phr, the much higher density of Gd_2_O_3_ (ρ = 7.4 g/cm^3^) than that of NR (approximately 0.93–0.97 g/cm^3^ [25]) resulted in the volume of Gd_2_O_3_ powder used during the compounding being much less than that of NR, making the mixing of all the chemicals on a two-roll mill possible. After the compounding, the NR samples were vulcanized using hot compression molding (CC-HM-2060, Chaicharoen Karnchang Co., Ltd., Bangkok, Thailand) at 150 °C and a pressure of 160 kg/cm^2^ for 150 secs in a mold with dimensions of either 15 cm × 15 cm × 0.2 cm or 10 cm × 10 cm × 0.2 cm. Notably, the procedure for sample preparation was mainly based on the published works of Xu et al. [23,24], while the cure time of 150 secs was selected following preliminary studies for optimized cure times, for which shorter or longer cure times resulted in the samples being too soft or too hard, respectively, which limited their useability and prevented the initiation of self-healing mechanisms from occurring [26].

### 2.3. Characterization

#### 2.3.1. Neutron-Shielding Properties

The neutron shielding properties of the Gd_2_O_3_/NR composites were investigated at the Thailand Institute of Nuclear Technology (Public Organization), Bangkok, Thailand. The neutron-shielding parameters investigated in this work were the neutron transmission (I/I_0_), the linear attenuation coefficient (µ), the half-value layer (HVL), and the tenth-value layer (TVL), and their relationships are shown in Equations (1)–(4) [14]:

(1) $$I/I_{0}={\;e}^{-\mu x}$$

(2) $$\mathrm{HVL}=\frac{\ln\left(2\right)}{\mu }$$

(3) $${µ}_{m}=\frac{\mu }{\rho }$$

(4) $$\mathrm{TVL}=\frac{\ln\left(10\right)}{\mu }$$

where I_0_ is the initial intensity of the incident neutrons, I is the final intensity of the transmitted neutrons, x is the thickness of the sample, and ρ is the density of the sample.

**Figure 2:**
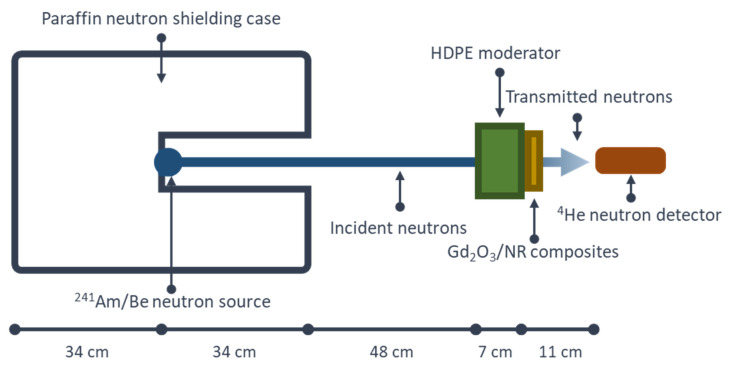
Schematic setup for neutron-shielding measurement.

The setup for neutron-shielding measurement is schematically shown in Figure 2, with a ^241^Am/Be used as a thermal neutron source. The values of I and I_0_ were recorded using a ^3^He neutron detector that was connected to a high-voltage supplier (Model 659, ORTEC, CA, USA), an amplifier (Model 2022, Canberra, CT, USA), and a time counter (Model TC 535P, Tennelec, TN, USA). The neutron source was positioned such that it was 0.89 m away from the NR sample and 1.00 m away from the detector. Notably, to investigate the effects of sample thickness on the neutron-shielding properties, the total thickness values of the NR samples were also varied (2, 4, 6, 8, and 10 mm).

#### 2.3.2. X-ray-Shielding Properties

**Figure 3:**
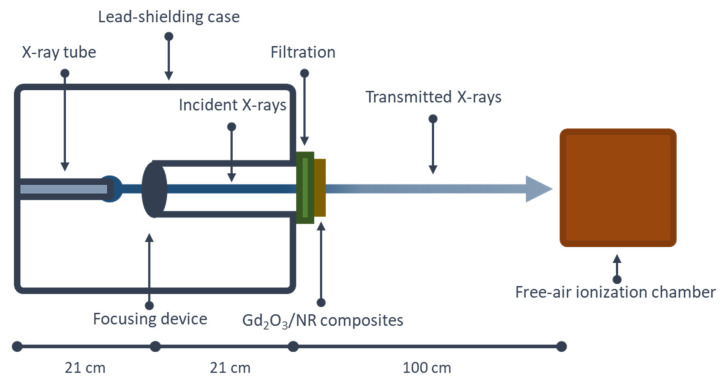
Schematic setup for X-ray-shielding measurement.

The schematic setup for X-ray-shielding measurement is shown in Figure 3. The measurement was carried out at the Secondary Standard Dosimetry Laboratory (SSDL), the Office of Atoms for Peace (OAP), Bangkok, Thailand. The X-ray-shielding parameters investigated in this work were X-ray transmission (I/I_0_), the linear attenuation coefficient (µ), the mass attenuation coefficient (µ_m_), the half-value layer (HVL), the tenth-value layer (TVL), and the Pb equivalence (Pb_eq_), for which Pb_eq_ could be determined using Equation (5) [14]:

(5) $${\mathrm{Pb}}_{\mathrm{eq}}=\frac{\mu x}{\mu_{\mathrm{Pb}}}$$

where µ_Pb_ is the linear attenuation coefficient of a pure Pb sheet. It should be noted that the values of µ_Pb_ were 63.06 cm^−^^1^ and 25.99 cm^−^^1^ for the incident X-ray energies of 45 keV and 80 keV, respectively, and were numerically determined using XCOM software (National Institute of Standards and Technology, Gaithersburg, MD, USA) [14,27]. The X-ray energies of 45 keV and 80 keV were selected for the determination of Pb_eq_ due to being the average energies of X-rays generated from an X-ray tube (YXLON MGC41, NY, USA) with the supplied voltages of 60 and 100 kV (Keithley 651B, OH, USA), respectively, used in this work. The emitted X-ray beam was collimated using a Pb collimator with a 1 mm pinhole, and the transmitted X-rays were detected and counted using a free-air ionization chamber (Korea Research Institute of Standards and Science; KRISS, Daejeon, Korea). More details for the setup of the neutron-shielding measurement are available in [14]. Similar to the neutron measurement, the total thickness values of the NR samples varied from 2 to 10 mm in 2 mm increments to investigate effects of material thickness on X-ray-shielding abilities.

#### 2.3.3. Mechanical Properties

The mechanical properties of tensile strength and elongation at break for all the Gd_2_O_3_/NR composites were determined using a universal testing machine (Auto-graph AG-I 5kN, Shimadzu, Kyoto, Japan) following ASTM D412-06 standard testing. The tensile testing speed used for all the samples was 50 mm/min. The surface hardness (Shore A) was determined using a hardness durometer (Teclock GS-719G, Japan) following the ASTM D2240-05 standard testing method.

For the determination of the self-healing capabilities of the developed Gd_2_O_3_/NR composites, samples having shapes and sizes based on ASTM D412-06 standard testing were cut into two equal pieces using a surgical knife and were immediately brough into contact. Then, after 60 min of contact, the samples were installed in a universal testing machine (Auto-graph AG-I 5kN, Shimadzu, Kyoto, Japan) to determine their tensile strength and elongation at break, following the same testing procedures as those for the uncut samples. Then, the tensile strength values of the self-healed samples were used for the calculation of the percentage of recoverable strength (%Recovery) using Equation (6) [18]:

(6) $$\%\mathrm{Recovery}=\frac{{\mathrm{TS}}_{\mathrm{self}-\mathrm{healing}}}{{\mathrm{TS}}_{\mathrm{uncut}}}\times 100\%$$

where TS_self-healing_ and TS_uncut_ are the tensile strengths of the self-healed and uncut samples, respectively [28].

#### 2.3.4. Density Measurement

The densities for all the Gd_2_O_3_/NR composites were determined using a densitometer (MH-300A, Shanghai, China) with a precision of 0.01 g/cm^3^, and the determination was carried out based on the Archimedes principle [29]. Additionally, to verify the correctness of the density measurement, theoretical densities (ρ_th_) for all the samples were calculated using Equation (7) [14]:

(7) $$\rho_{\mathrm{th}}=\frac{C_{\mathrm{NR}}+C_{\mathrm{Gd}2O3}}{\frac{C_{\mathrm{NR}}}{\rho_{\mathrm{NR}}}+\frac{C_{\mathrm{Gd}2O3}}{\rho_{\mathrm{Gd}2O3}}}$$

where C_NR_ is the content of NR, C_Gd2O3_ is the content of Gd_2_O_3_, ρ_NR_ is the density of NR (0.95 g/cm^3^), and ρ_Gd2O3_ is the density of Gd_2_O_3_ (7.4 g/cm^3^).

#### 2.3.5. Morphological Studies

The morphology, dispersion of Gd_2_O_3_ particles, and dispersion of Gd elements were determined using scanning electron microscopy (SEM) with energy-dispersive X-ray (EDX) spectroscopy (Quanta 450 FEI: JSM-6610LV, Eindhoven, the Netherlands) at a 10 kV accelerating voltage. Prior to the SEM-EDX studies, all specimens were coated with gold using a sputter coater (Quorum SC7620: Mini Sputter Coater/Glow Discharge System, Nottingham, UK) at a power voltage of 10 kV and a current of 10 mA for 120 secs.

## 3. Results and Discussion

### 3.1. Density

Table 2 indicates the experimental and theoretical densities, as well as the differences between these two values, of all the Gd_2_O_3_/NR composites investigated in this work. The results showed that the densities of the NR samples increased with increasing Gd_2_O_3_ content, while the differences between the experimental and theoretical values were below 5.0% for all the samples, clearly verifying the correctness and reliability of the experimental values for further use. The positive relationship between density and filler content was due to the much higher density of Gd_2_O_3_ than NR, resulting in a greater sample mass (determined at the same total volume) and, subsequently, greater overall density of the NR composites containing higher Gd_2_O_3_ contents [30].

### 3.2. Neutron-Shielding Properties

**Figure 4:**
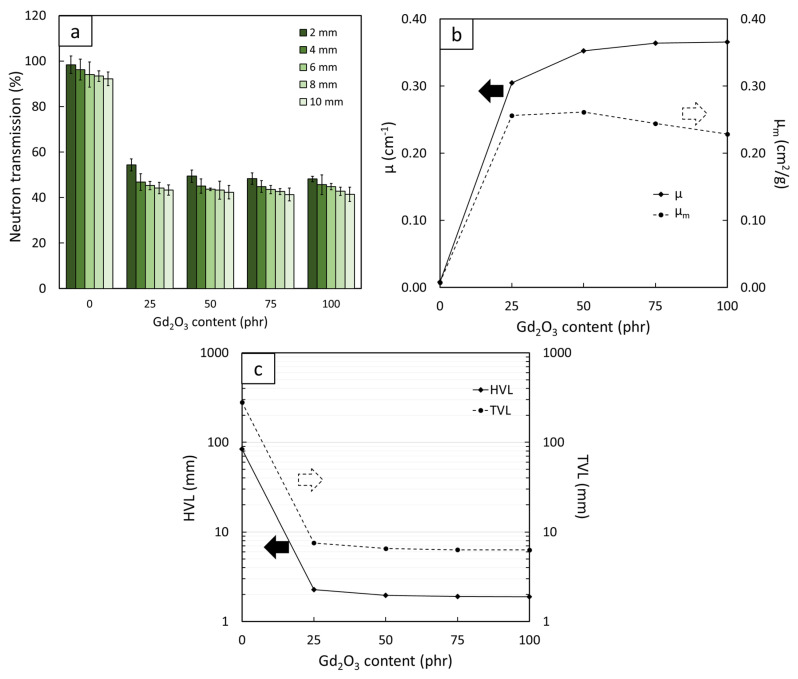
Neutron-shielding properties: (**a**) the neutron transmission, (**b**) linear attenuation coefficient (µ), mass attenuation coefficient (µ_m_), (**c**) half-value layer (HVL) and tenth-value layer (TVL) of Gd_2_O_3_/NR composites containing varying Gd_2_O_3_ contents of 0, 25, 50, 75, and 100 phr, where error bars indicate ± standard error.

**Figure 5:**
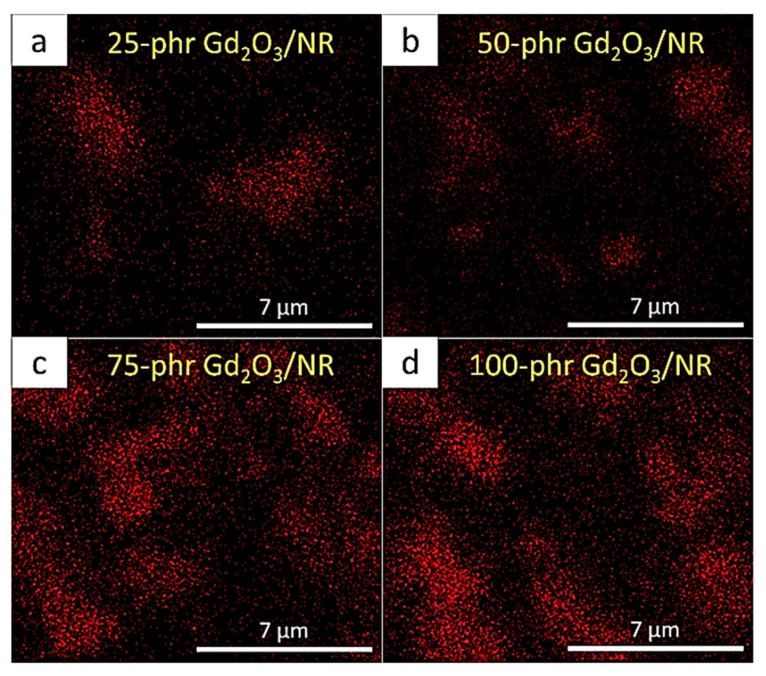
Dispersion of Gd_2_O_3_/NR composites containing varying Gd_2_O_3_ contents captured using SEM-EDX: (**a**) 25 phr, (**b**) 50 phr, (**c**) 75 phr, and (**d**) 100 phr.

The results for the neutron-shielding properties, consisting of I/I_0_, µ, µ_m_, HVL, and TVL, for all the Gd_2_O_3_/NR composites are shown in Figure 4, which indicates that the overall neutron-shielding properties of the samples increased with increasing Gd_2_O_3_ content, as evidenced by the lower values of I/I_0_, HVL, and TVL and the higher values of µ and µ_m_ in the samples containing higher contents of Gd_2_O_3_. This shielding enhancement from the addition of Gd_2_O_3_ was mainly due to Gd having a much higher σ_abs_ value than the C and H in NR (σ_abs_ values for Gd, C, and H are 49,700 barns, 0.0035 barns, and 0.3326 barns, respectively [31]), resulting in considerably increased chances for incident thermal neutrons to be absorbed and attenuated by the composites and, consequently, leading to superior neutron-shielding properties for the Gd_2_O_3_/NR composites [32]. Figure 5 shows the dispersion of Gd in the NR composites based on SEM-EDX and reveals that the highest elemental density of Gd was in the sample containing 100 phr Gd_2_O_3_ (Figure 5d), confirming the rationale for the improved neutron-shielding properties of the Gd_2_O_3_/NR composites by the addition of Gd_2_O_3_.

In addition, Figure 4 indicates that a small addition of 25 phr Gd_2_O_3_ to pristine NR could sharply increase the neutron-shielding properties of the samples, as seen by the sharp decreases in I/I_0_ and HVL from 97% and 84 mm in pristine NR to just 55% and 2.3 mm, respectively, in the 25 phr Gd_2_O_3_/NR sample (values of I/I_0_ and HVL were compared using 2 mm thick samples). This notable improvement in the neutron-shielding properties was mainly due to the sudden change in dominant neutron interactions from elastic scattering in the pristine NR to neutron absorption in the Gd_2_O_3_/NR composites, for which the latter mechanism was relatively more effective in neutron attenuation than the former [33]. However, as more Gd_2_O_3_ powder was added to the composites, only a slight improvement was observed, perhaps because the samples already relied on the preferable absorption mechanism such that further addition of Gd_2_O_3_ could only slightly increase the probabilities of neutrons being absorbed [34]. 

Another point worth mentioning is that the ability to attenuate neutrons increased with increasing sample thickness. The dependence of neutron-shielding properties on sample thickness, as illustrated in the determination of I/I_0_ and shown in Figure 4a, was mostly due to more materials being available to elastically scatter (in the case of a pristine NR sample) or absorb (in the case of Gd_2_O_3_/NR samples) incident neutrons in thicker samples, subsequently reducing the transmitted neutrons (lower I/I_0_). This relationship could also be mathematically explained using Equation (1) when re-arranged as shown in Equation (8), which depicts that the value of ln(I/I_0_) was inversely proportional to x (thickness of the sample); hence, I/I_0_ was negatively related to x:

(8) $$\ln\left(I/I_{0}\right)=-\mu x$$

### 3.3. X-ray-Shielding Properties

**Figure 6:**
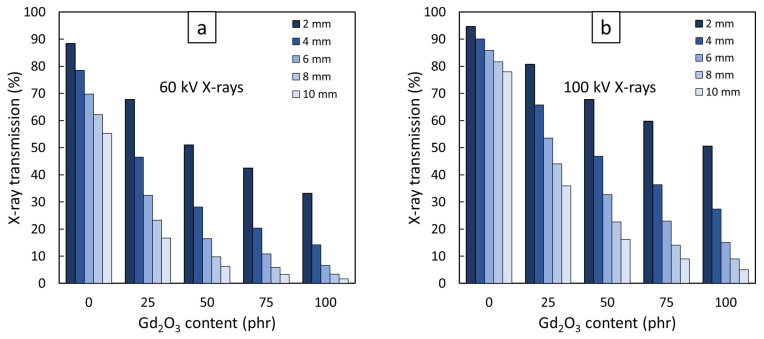
X-ray transmission of Gd_2_O_3_/NR composites with varying thicknesses from 2 to 10 mm and varying Gd_2_O_3_ contents from 0 to 100 phr determined at X-ray supplied voltages of (**a**) 60 kV and (**b**) 100 kV.

Figure 6 shows the results for the percentage of X-ray transmission for 60 kV and 100 kV X-rays, respectively, through varying thicknesses (2, 4, 6, 8, and 10 mm) of Gd_2_O_3_/NR composites. Similar to the results from the neutron-shielding measurement, the X-ray transmission decreased with increasing Gd_2_O_3_ content and sample thickness. The dependence of the X-ray transmission on Gd_2_O_3_ content was mainly due to the increased interaction probabilities between the incident X-rays and the materials through photoelectric absorption and Compton scattering with the addition of Gd_2_O_3_, for which their cross-sections were positively correlated to the Z and ρ values of the materials, hence improving the attenuation ability of the composites [35,36]. In addition, increasing the thickness of the samples could decrease X-ray transmission due to more Gd_2_O_3_ being available to interact with the incident X-rays, resulting in fewer X-rays being transmitted through the samples.

**Figure 7:**
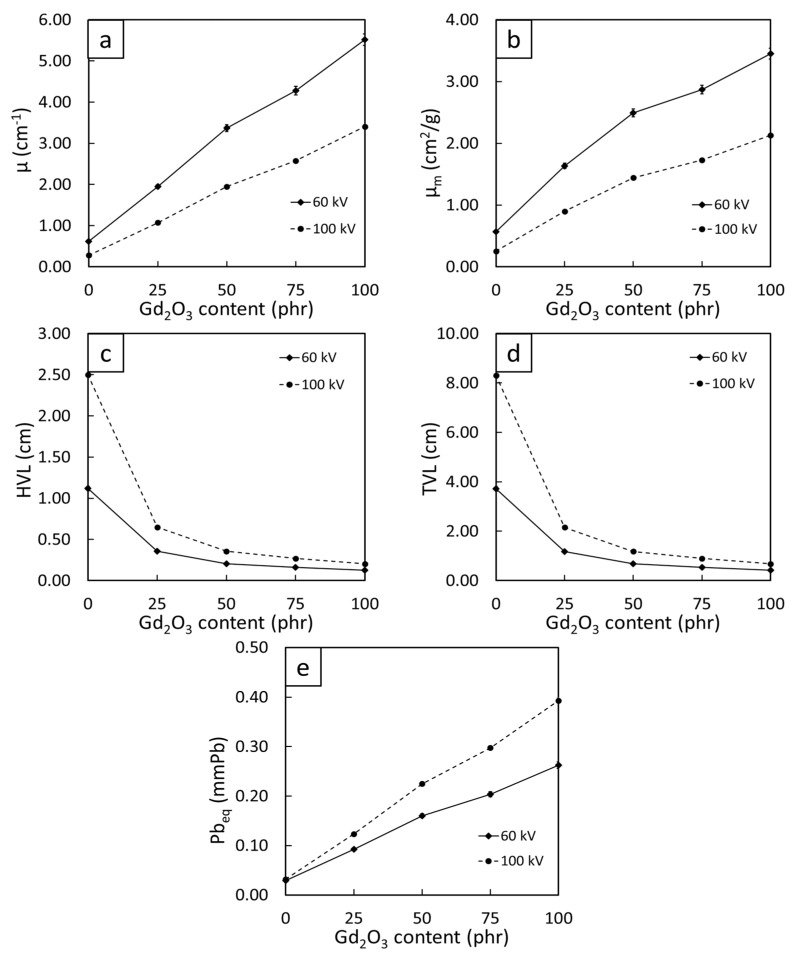
X-ray-shielding properties: (**a**) linear attenuation coefficient (µ), (**b**) mass attenuation coefficient (µ_m_), (**c**) half-value layer (HVL), (**d**) tenth-value layer (TVL), and (**e**) lead equivalence (Pb_eq_) of 3 mm thick Gd_2_O_3_/NR composites containing varying Gd_2_O_3_ contents of 0, 25, 50, 75, and 100 phr.

Figure 7 shows the results of the µ, µ_m_, HVL, TVL, and Pb_eq_ values of Gd_2_O_3_/NR composites containing varying Gd_2_O_3_ content determined using 60 kV and 100 kV X-rays (common supplied voltages used for medical diagnostics [37]). The results imply that the overall X-ray-shielding properties of the composites generally increased with increasing Gd_2_O_3_ content, as evidenced by the lowest values of HVL and TVL and the highest values of µ, µ_m_, and Pb_eq_ being found in the sample with 100 phr Gd_2_O_3_. For example, the values of HVL (Pb_eq_) of the NR composites were reduced from 1.12 cm and 2.50 cm, respectively, (0.03 mmPb and 0.03 mmPb) for pristine NR to 0.36 and 0.65 cm (0.09 mmPb and 0.12 mmPb) for 25 phr Gd_2_O_3_/NR composites, determined at 60 kV and 100 kV X-rays, respectively, exhibiting approximately a 3–4-fold improvement in X-ray attenuation ability with the addition of just 25 phr Gd_2_O_3_.

Another interesting result from Figure 7 was that the X-ray-shielding properties of the samples determined at the 60 kV supplied voltage were higher than those at the 100 kV supplied voltage due to the cross-section of the dominant photoelectric absorption (σ_pe_) being inversely related to the cube of incident X-ray energy (E), as shown in Equation (9) [38]:

(9) $$\sigma_{\mathrm{pe}}\propto \frac{Z^{n}}{E^{3}}$$

Consequently, the incident X-rays emitted from an X-ray tube with higher supplied voltages could have fewer chances of interaction with the materials, resulting in more X-rays being transmitted and, subsequently, lower overall X-ray attenuation properties than lower-energy X-rays [39]. Notably, the values of Pb_eq_ at a specific Gd_2_O_3_ content could be tailored according to the shielding requirements for intended applications by lowering or increasing the thickness of the sample (the sample thickness for Figure 7e was 3 mm) using Equation (5).

### 3.4. Comparative Neutron- and X-ray-Shielding Properties between Current and Other Similar Materials

Table 3 shows a comparison of neutron- and X-ray-shielding properties (based on the values of HVL) from this work with other similar materials, which indicates that the current materials exhibited comparable or better neutron and X-ray attenuation capabilities than those from other reports. As a result, this comparison clearly confirmed the useability and potential of the NR composites for utilization as effective neutron- and X-ray-shielding materials with potential self-healing capabilities. It should be noted that the differences in the HVL values from all the materials in Table 3 could be due to several factors, such as differences in the filler types and contents used, as well as various energies of the incident radiation during measurement.

### 3.5. Mechanical Properties

**Figure 8:**
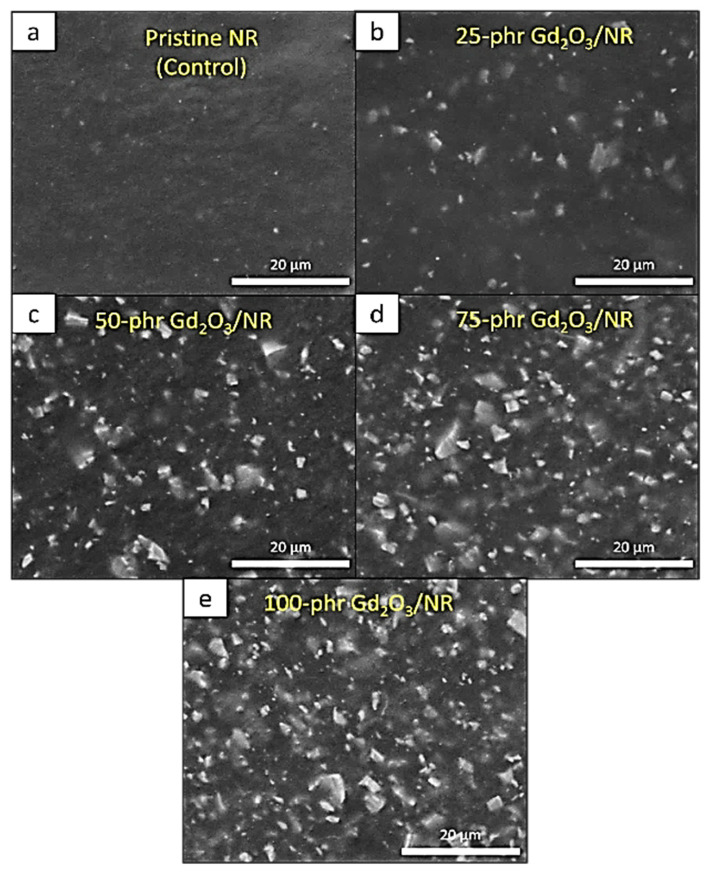
SEM images showing morphology and particle dispersion of NR composites containing varying Gd_2_O_3_ contents: (**a**) 0, (**b**) 25 phr, (**c**) 50 phr, (**d**) 75 phr, and (**e**) 100 phr.

Table 4 shows the mechanical properties—tensile strength, elongation at break, and hardness (Shore A) of Gd_2_O_3_/NR composites containing varying Gd_2_O_3_ contents (0, 25, 50, 75, and 100 phr). The results indicate that the values of tensile strength and elongation at break generally decreased, while the hardness (Shore A) increased with increasing Gd_2_O_3_ content. The decreases in tensile properties after the addition of Gd_2_O_3_ could be due to poor surface compatibility between Gd_2_O_3_ and the NR matrix, which possibly resulted in the formation of voids and discontinuities in the matrix, subsequently obstructing the transfer of external forces and reducing the overall strength and elongation of the materials [42]. Furthermore, the addition of high levels of Gd_2_O_3_ contents, especially at 100 phr, led to high agglomeration of the Gd_2_O_3_ particles due to filler–filler interactions that prevented more preferable and stronger rubber–filler interactions from occurring [43]. The SEM images depicting the dispersion of Gd_2_O_3_ particles in the NR matrix are shown in Figure 8 and reveal that, while the particles were fairly evenly distributed throughout the NR matrix, some particle agglomeration was found in samples with higher filler contents, especially at 50, 75, and 100 phr (Figure 8c–e), compared to pristine NR (Figure 8a) and the sample with 25 phr filler content (Figure 8b). On the other hand, hardness (Shore A) had a strong positive relationship with Gd_2_O_3_ content due to the high rigidity of the Gd_2_O_3_ particles, which enhanced the overall rigidity and, hence, the hardness of the composites [44]. These findings are consistent with other reports, where the mechanical properties of materials generally decrease with the addition of high filler content, especially those developed for use in radiation protection [13,14].

Comparing the tensile properties obtained from this work with another work by Xu et al. indicated that the current pristine NR samples were approximately four times higher in tensile strength, as evidenced by the values reported in [23,24] being lower than 2 MPa for all the formulations. The differences in mechanical properties between these two works could be due to several factors, such as different formulation and cure times, as well as the types of NR used for sample preparation, which can affect the degree of cross-linking and, hence, the strength of the composites [45].

### 3.6. Self-Healing Properties

**Figure 9:**
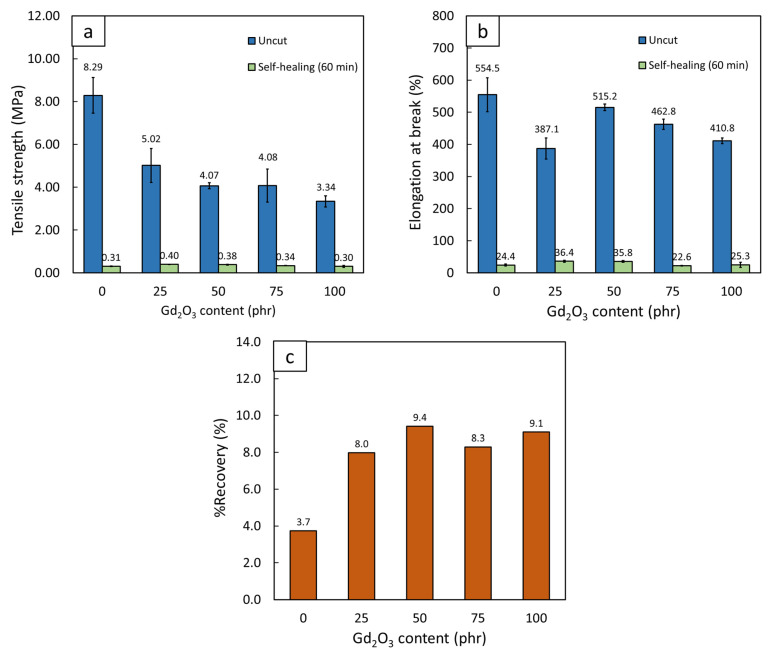
Comparison of (**a**) tensile strength, (**b**) elongation at break, and (**c**) percentage of recovery (%Recovery) for uncut and self-healed Gd_2_O_3_/NR composites containing varying Gd_2_O_3_ contents (0, 25, 50, 75, and 100 phr), where error bars indicate ± standard deviation.

Figure 9 shows the comparative values of strength and elongation at break, as well as the percentage of recoverable strength (%Recovery), of the original and 60 min self-healed NR composites containing varying Gd_2_O_3_ contents (0, 25, 50, 75, and 100 phr). The results indicate that the values of tensile strength and elongation at break for all the self-healed samples (Figure 9a,b) were lower than the original ones, with values for recoverable strength and elongation at break in the ranges of 0.30–0.40 MPa and 22.6–36.4%, respectively, leading to values of %Recovery in the range of 3.7–9.4% (Figure 9c).

The reduction in the tensile strength and elongation at break of the self-healed NR composites in comparison to the original samples could be explained by the NR molecular chains in the uncut samples being originally cross-linked with a combination of covalent and ionic bonds, which resulted in relatively high tensile strength and elongation at break before the cut [46]. However, as the two damaged surfaces were gently pressed together and remained in contact for 60 min at room temperature (approximately 25 °C), a reversible ionic supramolecular network via the polymerization of ZDMA was able to recreate the sample through the mobility of NR molecular chains and, subsequently, restore some recoverable strength to the self-healed surfaces [47]. Nonetheless, the overall strengths of the self-healed samples were considerably lower than the original samples, with %Recovery values in the range of 3.7–9.1% (Figure 9c). This could be due to the reduced level of cross-link density in the samples after self-healing that could only be recreated by ionic bonds. On the other hand, the covalent bonds, which also initially presented and played major roles in providing exceptional strength to the original samples, were irreversible and, consequently, absent in the self-healed contact, resulting in much-reduced levels of recoverable strength and elongation at break for the samples [48]. Another factor that affected the self-healing mechanism was the addition of the Gd_2_O_3_ particles to the NR matrix, for which the fillers were not a part of the reversible supramolecular network and, hence, hindered or blocked the initiation of self-healing [24]. 

Nonetheless, despite having Gd_2_O_3_ contents of up to 100 phr, the recoverable strengths of the Gd_2_O_3_/NR composites were in the range of 0.30–0.40 MPa, which were in the same order of magnitude as that of pristine NR reported by Xu et al. (being in the range of 0.5–0.7 MPa, depending on self-healing times [23]), implying the useability and potential of the current self-healing materials for applications in radiation protection. Furthermore, the success of the current work could promote further attempts to develop ‘smart’ and more effective materials for use in radiation shielding, along with the previously reported composites of Bi_2_O_3_/PVA, Sm_2_O_3_/PVA, Gd_2_O_3_/PVA, graphene/PVA, and PbO_2_/acrylamide [18,28,49,50]. It should be noted that the %Recovery values of the NR samples with the addition of Gd_2_O_3_ were higher than that of the pristine NR because the original Gd_2_O_3_/NR composite had 2–3 times lower tensile strength than pristine NR, while having similar recoverable strength after self-healing, which resulted in considerably higher %Recovery values for the Gd_2_O_3_/NR composites.

## 4. Conclusions

This work developed dual neutron-shielding and X-ray-shielding NR composites containing varying contents of Gd_2_O_3_ (0, 25, 50, 75, and 100 phr) with autonomously self-healing capabilities. The results showed that the added Gd_2_O_3_ acted as an effective protective filler against neutrons and X-rays, as evidenced by the decreases in I/I_0_, HVL, and TVL and the increases in µ, µ_m_, and Pb_eq_ of the NR composites after being added to the matrix. In addition, the results indicated that the increased filler content led to decreased tensile strength and elongation at break, whereas the hardness (Shore A) increased, mainly due to the initiation of particle agglomeration at high filler contents and poor surface compatibility between the NR matrix and the filler. The developed NR composites also offered self-healing capabilities at the fractured surfaces through a reversible ionic supramolecular network, with recoverable strength and %Recovery values in the ranges 0.30–0.4 MPa and 3.7–9.4%, respectively (after self-healing for 60 min). Based on the overall results obtained, the developed Gd_2_O_3_/NR composites showed great potential for use as novel and self-healing radiation-shielding materials that could effectively attenuate both neutrons and X-rays, thus prolonging the lifetime of the protective material and enhancing the safety for users, as well as being a basis for the future development of ‘smart’ shielding materials.

## Figures and Tables

**Table 1:** Material formulations of Gd_2_O_3_/NR composites and their chemical names, contents, roles, and suppliers.

Chemical	Content (phr)	Role	Supplier
Natural rubber (NR: STR 5CV)	100	Main matrix	Hybrid Post Co., Ltd. (Bangkok, Thailand)
Zinc dimethacrylate (ZDMA)	40	Accelerator	Shanghai Ruizheng Chemical Technology Co., Ltd. (Shanghai, China)
Dicumyl peroxide (DCP)	1	Curing agent	Shanghai Ruizheng Chemical Technology Co., Ltd. (Shanghai, China)
Gadolinium oxide (Gd_2_O_3_)	0, 25, 50, 75, and 100	Radiation-protective filler	Shanghai Ruizheng Chemical Technology Co., Ltd. (Shanghai, China)

**Table 2:** Experimental and theoretical densities, as well as the differences between the methods, of Gd_2_O_3_/NR composites with varying Gd_2_O_3_ contents of 0, 25, 50, 75, and 100 phr. Experimental densities shown as mean ± standard deviation.

Gd_2_O_3_ Content (phr)	Experimental Density (g/cm^3^)	Theoretical Density (g/cm^3^)	Difference (%)
0 (Control)	0.99 ± 0.01	0.95	4.0
25	1.19 ± 0.01	1.15	3.4
50	1.35 ± 0.01	1.33	1.5
75	1.49 ± 0.01	1.51	1.3
100	1.60 ± 0.01	1.68	5.0

**Table 3:** Comparison of neutron- and X-ray-shielding properties based on the half-value layer (HVL) between the results from this work and those from similar materials. Numbers in parentheses represent supplied voltages used for X-ray-shielding measurement.

Main Matrix	Filler	Filler Content	Half-Value Layer (mm)		Reference
			Neutrons	X-rays	
NR	Gd_2_O_3_	50 phr	2.0	2.1 (60 kV)/3.6 (100 kV)	This work
NR	Gd_2_O_3_	75 phr	1.9	1.6 (60 kV)/2.7 (100 kV)	This work
NR	B_2_O_3_	80 phr	3.2	–	[11]
PVA	Sm_2_O_3_	10.5 wt%	4.2	–	[18]
PVA	Gd_2_O_3_	10.5 wt%	3.6	–	[18]
EPDM	B_2_O_3_	42.6 phr	3.7	–	[40]
NR	Bi_2_O_3_	50 phr	–	6.0 (60 kV)	[14]
NR	BaSO_4_	50 phr	–	6.0 (60 kV)	[14]
NR	Bi_2_O_3_	40 phr	–	~3.5 (120 kV)	[41]
NR	Bi_2_O_3_	80 phr	–	~3.0 (120 kV)	[41]

**Table 4:** Mechanical properties of tensile strength, elongation at break, and hardness (Shore A) of Gd_2_O_3_/NR composites containing varying Gd_2_O_3_ contents of 0, 25, 50, 75, and 100 phr. Values are shown as mean ± standard deviation.

Gd_2_O_3_ Content (phr)	Tensile Strength (MPa)	Elongation at Break (%)	Hardness (Shore A)
0 (Control)	8.29 ± 0.83	555 ± 53	38 ± 1
25	5.02 ± 0.79	387 ± 33	41 ± 1
50	4.07 ± 0.14	515 ± 10	45 ± 1
75	4.08 ± 0.77	463 ± 16	46 ± 1
100	3.34 ± 0.26	411 ± 9	50 ± 1

## Data Availability

The data presented in this study are available on request from the corresponding author.
